# Long-term effect of a four-drugs induction regimen for patients with high baseline viral load

**DOI:** 10.7448/IAS.17.4.19776

**Published:** 2014-11-02

**Authors:** Franco Maggiolo, Giulia Masini, Noemi Astuti, Elisa Di Filippo, Simone Benatti, Daniela Valenti, Anna Paola Callegaro, Marco Rizzi

**Affiliations:** 1 Division of Infectious Diseases, AO Papa Giovanni XXIII BergamoBergamo, Italy; 2 Laboratory of Virology and Microbiology, AO Papa Giovanni XXIII Bergamo, Bergamo, Italy

## Abstract

**Introduction:**

The long-term effects of an intensified induction regimen are unknown. In this pilot, randomized, prospective study we evaluate the effect of a short-term four-drugs induction regimen in patients with high baseline viral load.

**Methods:**

Naive patients with HIV-RNA>100.000 copies/ml receiving TDF+FTC+EFV+RAL (group ER) for 4 months and were then simplified to TDF+FTC+EFV. Two randomized control groups treated *ab-initio* with TDF+FTC+EFV (E) or TDF+FTC+RAL (R) were used.

**Results:**

19 patients with a mean age of 38 years and mean baseline CD4 count of 334 (SD 216) cells/mcL and HIV-RNA of 5.47 log (SD 0.32) copies/mL were enrolled. No baseline significant difference was observed among groups. Early HIV-RNA reduction was significantly higher in ER compared to the other groups from week 1 to week 4 (P from 0.026 to 0.003) (figure 1), thereafter HIV-RNA values were comparable among the groups. At week 96, all patients had an HIV-RNA < 50 copies/mL, however only patients in the ER group had in all cases an HIV-RNA level < 3 copies/mL with a statistically significant difference compared to E (60%; P=0.038) and R (50%; P=0.020). At 96 weeks, CD4 cell median counts were 765 cells/mcL for ER, 600 cells/mcL for E and 771 for R (P=0.16), however patients in the ER group presented a lower proport**i**on of activated CD4+CD38+HLADR+ cells (1.9% versus 3.9 and 3.8%) and CD8+CD38+HLADR+ cells (10.3% versus 16.8 and 16.5%) and a significantly better CD4/CD8 ratio (0.98 versus 0.53 and 0.61; P=0.03).

**Conclusions:**

A four-drug regimen in naive patients with high pre-therapy viral load improves early virologic response. A quick drop of HIV-RNA seems to correlate with a sustained virologic response. Although limited in time (four months), the four-drug regimens correlates with an improved immunological response as measured by the CD4/CD8 ratio or the percentage of activated CD4+ and CD8+ cells. The reasons why this happens deserve further studies. This study highlights the importance of a personalised therapy especially in high risk patients.

**Figure 1:**
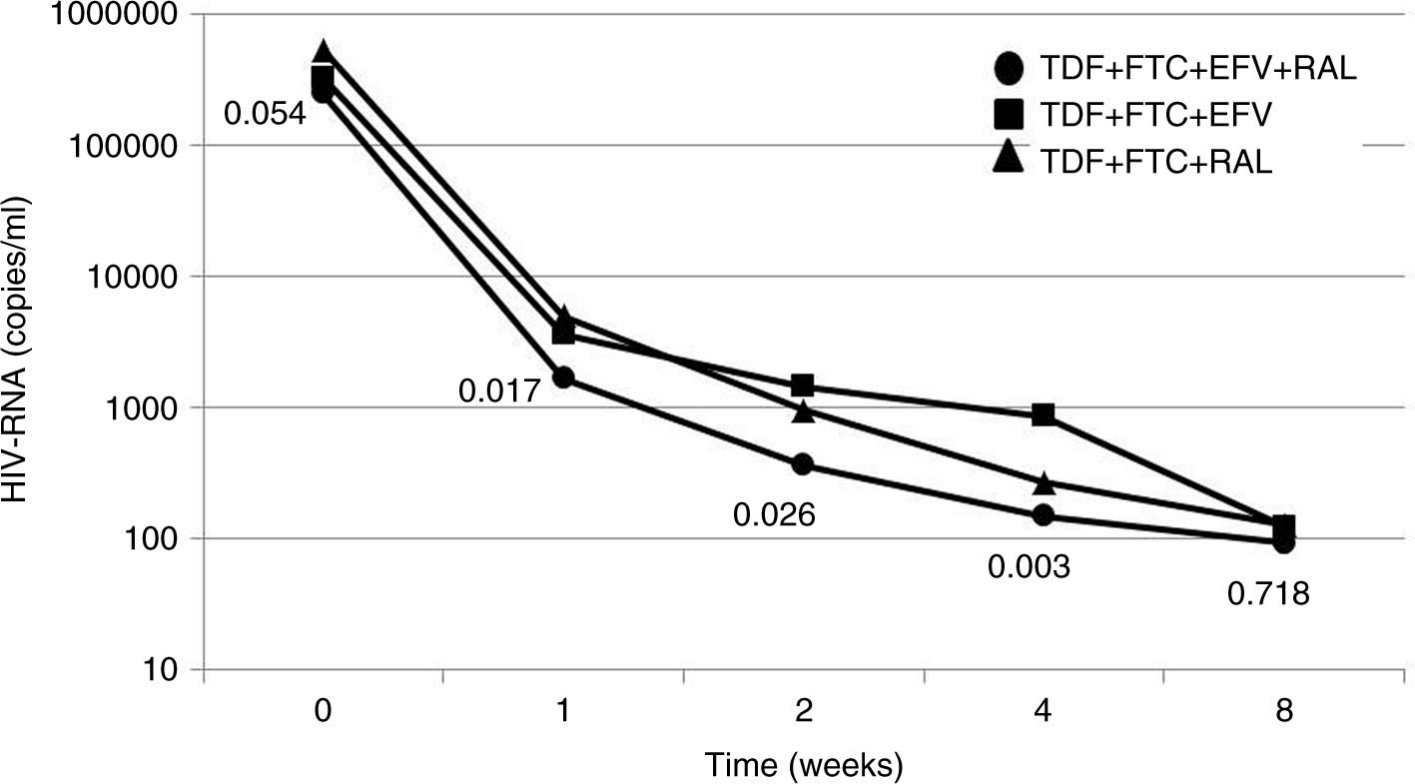
HIV-RNA reduction among the three groups.
